# Conventional Training Integrated with SteamVR Tracking 2.0: Body Stability and Coordination Training Evaluation on ICAROS Pro

**DOI:** 10.3390/s25092840

**Published:** 2025-04-30

**Authors:** Katharina Meiszl, Fabian Ratert, Tessa Schulten, Daniel Wiswede, Lara Kuhlmann de Canaviri, Tobias Potthast, Marc Silberbach, Laurin Hake, Yannik Warnecke, Witold Schiprowski, Mathias Merschhemke, Christoph M. Friedrich, Raphael Brüngel

**Affiliations:** 1 Department of Computer Science, University of Applied Sciences and Arts Dortmund (FH Dortmund), 44227 Dortmund, Germany; 2 Department of Medical Informatics, Biometry and Epidemiology, Ruhr University Bochum, 44789 Bochum, Germany; 3 Turn- und Sport-Club Eintracht von 1848/95 Korporation zu Dortmund (TSC Eintracht Dortmund), 44139 Dortmund, Germany; 4 Institute for Medical Informatics, Biometry and Epidemiology (IMIBE), University Hospital Essen, 45122 Essen, Germany; 5 Institute for Artificial Intelligence in Medicine (IKIM), University Hospital Essen, 45131 Essen, Germany

**Keywords:** body stability and coordination, ICAROS Pro, SteamVR Tracking 2.0, app-guided training, virtual reality, exergame

## Abstract

Technological advances continually reduce the effort to digitally transform health-related activities such as rehabilitation and training. Exemplary systems use tracking and vital sign monitoring to assess physical condition and training progress. This paper presents a system for body stability training and coordination evaluation, using cost-efficient tracking and monitoring solutions. It implements the use case of app-guided back posture tracking on the ICAROS Pro training device via SteamVR Tracking 2.0, with pulse and respiration rate monitoring via Zephyr BioHarness 3.0. A longitudinal study on training effects with 20 subjects was conducted, involving a representative procedure created with a sports manager. Posture errors served as the main progress indicator, and pulse and respiration rates as co-indicators. Outcomes suggest the system’s capabilities to foster comprehension of effects and steering of exercises. Further, a secondary study presents a self-developed VR-based exergame demo for future system expansion. The Empatica EmbracePlus smartwatch was used as an alternative for vital sign acquisition. The user experiences of five subjects gathered via a survey highlight its motivating and entertaining character. For both the main and secondary studies, a thorough discussion elaborates on potentials and current limitations. The developed training system can serve as template and be adjusted for further use cases, and the exergame’s reception revealed prospective extension directions. Software components are available via GitHub.

## 1. Introduction

The past decade has been defined by a digital transformation in healthcare. This was facilitated by adoption of further advancing and newly emerging technologies, such as artificial intelligence, sensory analytics, telemedicine, smart devices/wearables, and mobile health applications. These enable a transition towards more secure and qualitative healthcare [1,2,3,4]. The expansion of conventional therapy, including diagnostic and therapeutic tools/systems, has the potential to reduce costs as well as to enhance access to medical services and personnel [5,6,7,8]. Notable innovations include the adoption of low-cost sensor systems such as virtual reality (VR)-based tracking systems derived from the gaming industry. Use cases include training and rehabilitation concepts that utilize tracking for mixed reality (MR) applications, which combine physical and virtual elements [9,10,11]. One example may be serious games, which engage players in an entertaining and effective way to achieve specific goals, such as training and rehabilitation outcomes or behavioral changes. In the context of healthcare, these are being utilized increasingly to promote motivation, engagement, and the general sustainability of habits with beneficial effects on one’s health [12,13,14,15].

The preliminary work [16] on which this study is based presented a feasibility study on such a system. There, mobile application (app)-guided body stability training and coordination evaluation for rehabilitative purposes was established by coupling a conventional analog training device with low-cost VR tracking and vital sign sensors [16]. Prior to this, other preliminary work [17] evaluated whether the used tracking framework is suitable for such and further use cases. An initial evaluation of the functionality of the system demonstrated that it allows for measurable and reproducible execution of training procedures as well as enhanced comprehension of training progress.

The paper at hand addresses the continued development and thorough evaluation of said app-guided training system, which was modified for the use case of regular training. It is based on an ICAROS Pro training device, integration of back posture tracking via the low-cost SteamVR Tracking 2.0 framework and hardware, and vital sign monitoring via Zephyr BioHarness 3.0. The training system is designed to enhance body stability and coordination, facilitating quantifiable and reproducible training. This study demonstrates a series of training sessions with n=20 subjects and evaluates the training progress. Another potential application of the training system is a VR-based exergame, which is evaluated as part of a series of gaming sessions with n=5 subjects in an exploratory study aimed to gather initial user feedback. The main contributions of this work comprise the following:

- A free and open-source software platform for the inclusion of SteamVR Tracking 2.0 into conventional training procedures on analog training devices.
- A platform-connected app for user guidance and feedback during the execution of freely configurable training procedures.
- A thorough evaluation of the training system and investigation of training effects for the use case of body stability and coordination training, with a representative training procedure conceptualized by a sports manager.
- A showcase for further extension of the platform towards VR-based exergaming, teasing directions of future work.

The manuscript contents are organized as follows: Section 2 describes related work. Section 3 illustrates the materials and methods used, including the main components of the training system and the VR-based exergame showcase, as well as the demographics of the two study populations. Section 4 explains the design and implementation of the two use cases and the methodology for subsequent evaluations. Evaluation results are reported in Section 5, followed by a discussion for both evaluations in Section 6, highlighting current limitations. Section 7 then concludes with perspectives on the system and plans for further extension in future work.

## 2. Related Work

In regard to sensor-based training systems, related work has focused on the development of training systems incorporating tracking technologies into established therapeutic or preventive interventions in the context of cognitive and physical rehabilitation [18,19,20,21,22]. The purpose of integrating tracking technologies is to evaluate user movement in real time, thereby facilitating appropriate feedback on treatment outcomes. The results indicate feasible and effective approaches to improve patient treatment success. Expanding training systems with advanced training devices like the ICAROS Pro can increase motivation to engage in physical activity, thereby promoting physical fitness and health [23,24]. In further research, the ICAROS Pro was utilized to promote heightened interest and perceived attractiveness of the developed systems [25,26].

The research field of VR and exergames for rehabilitation and physical training shows that VR-based rehabilitation programs are more effective than traditional rehabilitation programs [27,28]. Other studies of exergames using VR have shown increased user satisfaction [29]. Additional studies have concluded that exergames can have an effect similar to moderate- to high-intensity exercise [30]. In addition, the perceived exertion is reduced due to the playful nature of exergames [30].

In the field of healthcare, the deployment of tracking technologies must align with established quality criteria to ensure suitability for clinical applications. In this regard, high position and orientation accuracy and low latency are of essential importance [31,32]. As a low-cost alternative to current leading professional tracking systems used in the industrial sector, the SteamVR tracking system, especially the latest version of the tracking system SteamVR Tracking 2.0, offers high accuracy in the sub-millimeter and sub-degree range [17,33,34]. In related work, SteamVR Tracking 2.0 is already being used in the field of training and rehabilitation systems [35,36,37,38,39,40,41].

Wearable technologies and sensor systems are increasingly utilized for recording and monitoring vital parameters during training and rehabilitation [42,43]. In previous own [16] and other [44,45,46] research, the Zephyr BioHarness 3.0 was used as a low-cost wearable monitoring device for vital signal acquisition. It is well known for its high accuracy, precision, and reliability [47,48,49]. In particular, the measurements of pulse and respiration rates showed corresponding results with a small deviation of ±1 beats per minute (bpm) as well as respirations per minute (rpm) compared to a reference device [47,49]. Consequently, the Zephyr BioHarness 3.0 has been adequately used as the gold standard [50,51,52,53]. In further recent research [54,55,56], the EmbracePlus from Empatica showed potential as a reliable data acquisition tool. The EmbracePlus is designed as a smartwatch for continuous monitoring [57]. Especially the measurement of peripheral oxygen saturation ($SpO_{2}$) data was validated with an accuracy root mean square error of 2.4% with a correlation of (r=0.96, p<0.001) between the measurement of the EmbracePlus and the arterial oxygen saturation ($SaO_{2}$) as reference [58].

The effectiveness of head-mounted display (HMD) for VR exergaming was demonstrated for both training [59] and rehabilitation [29]. Modern HMDs show a downward trend in complaints about cybersickness and immersion [60]. The HTC VIVE Pro 2 is such an HMD and was used for the realization of exergames and serious games [61,62,63].

## 3. Materials and Methods

The following subsections state the used materials and methods for implementation of the training system, the exergame, and the evaluations carried out for both. This includes descriptions of employed hardware and software components, evaluation methodologies, characteristics of the study populations, and the signal processing.

### 3.1. ICAROS Pro

**Figure 1:**
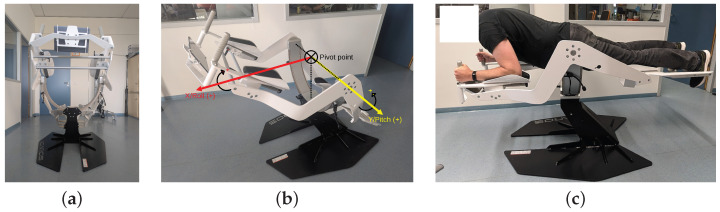
Different views of the ICAROS Pro training device. (**a**) Front view. (**b**) Side view with sketched axes. (**c**) Mounted by a 2 m tall person.

The ICAROS Pro (https://web.archive.org/web/20240706074919/https://www.icaros.com/en/products/icaros-pro/ (this and all other hyperlinks were, if not stated otherwise, last accessed on 29 January 2025)), shown in Figure 1, is a training device for isometric core strength plank exercises, allowing use of VR during training. It is used to train torso and upper body musculature, and targets the improvement of posture and coordination. During a plank, the body forms a line of head, shoulders, abdomen, pelvis, and legs while facing the floor. Support is given by forearms and elbows as well as knees and feet. A straight posture is to be maintained while rotating around the two axes of the ICAROS Pro, X/Roll and Y/Pitch, achieved by shifting weight. It has an adjustable length of 2.029 m–2.078 m, a width of 0.960 m, and a height of 0.960 m in its initial pose, and weighs 124 kg. The movement range amounts to ±35° on the X/Roll axis, and ±45° on the Y/Pitch axis around the pivot. Its safety was tested by TÜV Rheinland. An optional ICAROS Tablet Holder Universal [64] allows mounting of mobile devices with a screen diagonal of 4.5 inch–18 inch.

### 3.2. SteamVR Tracking 2.0

**Figure 2:**
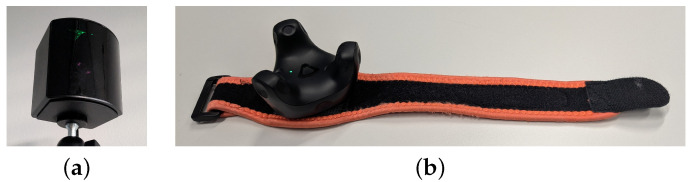
SteamVR Tracking 2.0 components: (**a**) one of four SteamVR Base Stations 2.0 on a mount, (**b**) one of two HTC VIVE Trackers (3.0) on a strap.

The SteamVR Tracking (https://partner.steamgames.com/vrlicensing) is a real-time tracking system with hardware and software components, developed for VR gaming purposes by Valve Corporation (https://www.valvesoftware.com/de/). The tracking system is based on the main components: a host, multiple base stations, and trackable hardware. The current version 2.0 has been available since 2018 and operates the optimized SteamVR Base Stations 2.0, (https://www.vive.com/de/accessory/base-station2) shown in Figure 2a. It is based on an inside-out principle in which the base stations alternately transmit 160° horizontal and 115° vertical infrared (IR) laser scans using sync-on-beam signals at a rate of 100 Hz. While using four base stations, an area of 10 m × 10 m can be covered. The surface of the trackable hardware, e.g., the VIVE Tracker (3.0) (https://www.vive.com/de/accessory/tracker3) in Figure 2b, is equipped with photodiodes to detect the base stations scans with Swept Angle Laser Tracking information. For the VIVE Tracker (3.0), the surface photodiodes allow a 240° field of view. The registered time differences enable the position and orientation estimation. Then, the estimated tracking information is transmitted from the trackable hardware to a host. The VIVE Tracker (3.0) is connected to the host via a Universal Serial Bus (USB) 2.0 interface using a dongle. The host is a SteamVR Tracking 2.0 framework, e.g., using Unity3D (https://unity3d.com/unity/whats-new/2021.1.25) version 2021.1.25 as the Application Programming Interface (API). The SteamVR plugin (https://assetstore.unity.com/packages/tools/integration/steamvr-plugin-32647) for Unity3D records the tracking information with an integration of SteamVR Tracking 2.0.

### 3.3. HTC VIVE Pro 2

**Figure 3:**
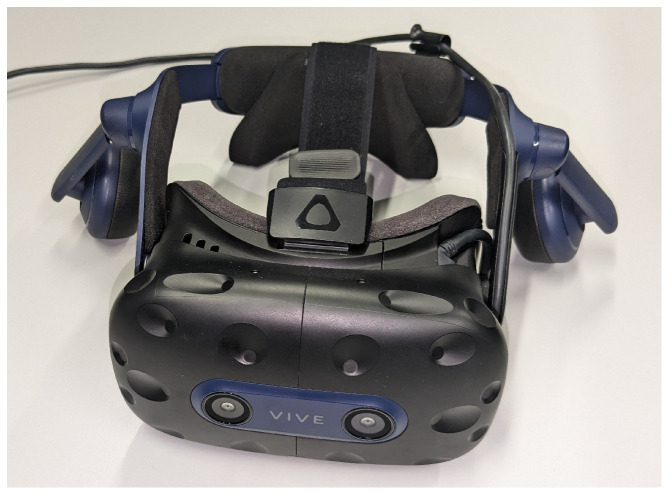
Top view of the HTC VIVE Pro 2.

For the VR component of the secondary study, the HTC VIVE Pro 2 (https://www.vive.com/de/product/vive-pro2/specs/) headset, shown in Figure 3, was used due to its high resolution and ergonomic design, providing an immersive and detailed user experience. The VIVE Pro 2 features dual LCD screens, each with a resolution of 2448 px × 2448 px per eye (4896 px × 2448 px in total), resulting in sharp visuals and reducing the screen door effect common in older VR headsets. The display supports 90 Hz and 120 Hz refresh rates. The field of view extends to 120°, allowing for a wide peripheral view in VR. The headset was paired with SteamVR Tracking 2.0 that provide 6 degrees of freedom tracking, allowing subjects to move freely within the ICAROS Pro with precise head tracking. The VR headset could be customized for subjects by adjusting the interpupillary distance between 57 mm and 70 mm. The headset was designed to be worn for extended periods of time with an adjustable headband and built-in weight distribution to minimize user fatigue. Audio was delivered through high-fidelity on-ear headphones, providing spatial audio that contributed to the immersive experience.

### 3.4. Zephyr BioHarness 3.0

**Figure 4:**
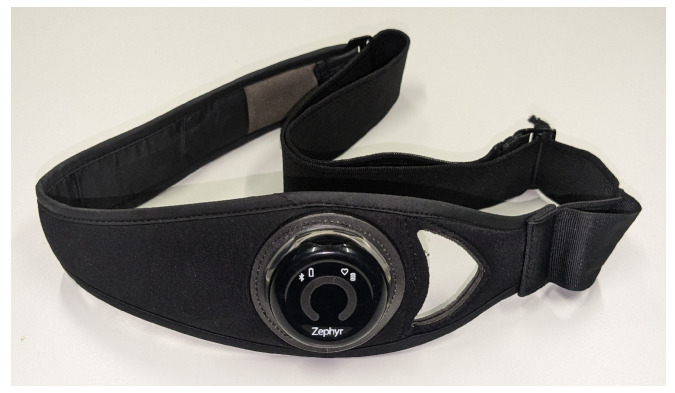
View of the Zephyr BioHarness 3.0.

The Zephyr BioHarness 3.0 (https://www.zephyranywhere.com/media/download/bioharness3-user-manual.pdf) for physiological monitoring, shown in Figure 4, consists of a sensor module attached to a chest strap. Measuring 28 mm in diameter and 7 mm in height, the 18 g light module collects, processes, stores, and transmits physiological information, including pulse and respiration rates. The chest strap weighs 71 g and is available in three sizes with different lengths. With a sampling rate of 250 Hz and a detection range of 25 bpm to 240 bpm, conductive pads capture an electrocardiogram (ECG). Pressure sensor pads detect respiratory chest expansion at a sampling rate of 25 Hz, ranging from 3 rpm to 70 rpm. This information is delineated in the official user manual, yet it should be minded that related work has also provided varying information with a sampling rate of 17 Hz [65]. Data transmission is via Bluetooth (version 2.1 + Enhanced Data Rate) using the proprietary Zephyr protocol.

### 3.5. Empatica EmbracePlus

**Figure 5:**
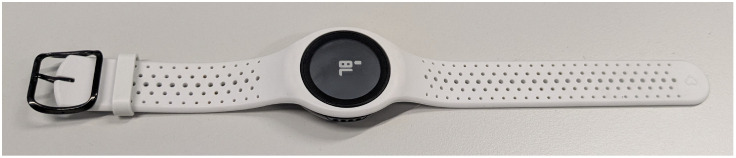
Top view of the Empatica EmbracePlus.

The EmbracePlus (https://www.empatica.com/en-eu/embraceplus/ (29 January 2025)), shown in Figure 5, is a smartwatch developed by Empatica Inc., Boston, MA, USA, and is CE-certified as a class IIa medical device and has a 510(k) U.S. Food and Drug Administration (FDA) clearance. In addition to recording ventral Electrodermal Activity (EDA) data, a photoplethysmogram sensor allows measurement of heart rate variability. Data recorded can be stored on the device and be transmitted via an encrypted Bluetooth 5.0 connection. The size of the EmbracePlus is 40 mm in width, 38.5 mm in length, and 11 mm in height. The wrist strap is adjustable, with a range of circumference of the smartwatch between 95 mm and 222 mm.

### 3.6. Study Populations

The study populations for the evaluations of the two applications are presented below. The main study focuses on the evaluation of the training system. The secondary study examines the applicability of the VR-based exergame. All experiments of both studies were conducted in concordance with national and international legislation as well as the Declaration of Helsinki.

The main study population consisted of a group of n=20 volunteers, recruited by word of mouth, all of whom completed the study. Informed consent for data acquisition and processing was obtained. Demographic data are listed in Table 1. Sexes were represented equally. Subjects showed a mean age of 27.95 years. The mean height and weight were 1.78 m and 84.05 kg, and the mean Body Mass Index (BMI) was 26.29. Self-assigned fitness was scored on a 1–5 Likert scale (unfit–fit). No subject reported a history of visual, cognitive, cardiovascular, neurological, or musculoskeletal conditions.

A group of n=5 subjects studying in the department of the university participated in the secondary VR-based study. The same acquisition procedure was applied as in the main study, and informed consent was obtained. Subjects were on average 26.00 years old, 1.78 m tall, and weighed 80.20 kg with a mean BMI of 24.41. Sexes were represented by 2 women and 3 men. Further characteristics are listed in Table 2. Four of the five subjects had prior experience with VR games or applications.

### 3.7. Evaluation Survey

A survey was used to evaluate the secondary study. This combined aspects such as general software solutions, VR applications, gamification influences, and physical training. ChatGPT 4.0 was consulted to select questions from established questionnaires for the topics user experience evaluation, VR assessment, and fitness program effectiveness. This resulted in integration of, e.g., elements from the System Usability Scale [66] and the User Experience Questionnaire [67]. For the evaluation of VR aspects, items were selected from the iGroup Presence Questionnaire [68] and the Simulator Sickness Questionnaire [69]. These allow the assessment of immersion and potential discomforts like motion sickness. In addition, questions related to perceived exercise intensity and effectiveness were inspired by the Borg Scale of Perceived Exertion [70].

The survey was originally created and provided in German; Table 3 provides concise translations of asked questions. The first part focused on demographic and anthropometric aspects, involving age, sex, height, and weight (not explicitly covered in Table 3). In addition, a self-assigned fitness level and previous experience with VR games or applications were collected. The second part focused on user experience, containing questions on experienced usability, immersion, and potential averse effects while using the exergame. In the third part, somatic aspects were addressed, which focused on training effectiveness, strained muscle regions, and perceived exhaustion. The fourth part aimed at the gamification aspects with respect to the experience of fun and motivation, and the perceived balance between challenges and rewards. Technical aspects were addressed in the fifth part, where the graphics quality was to be rated and experienced bugs could be reported. The sixth part covered perceived satisfaction and willingness to recommend the exergame to others. The last part involved general open questions on particularly enjoyed aspects, potential improvements for a better training experience, and general feedback or suggestions.

### 3.8. Signal Processing

In the main study, data were collected via a Bluetooth connection at a rate of one sample per second. Signals were continuously transmitted from the source device to a receiver unit, which recorded the raw data without any real-time pre-processing. These collected datasets were stored in a dedicated database at the end of each training session. In the secondary study, data were collected using an Empatica EmbracePlus and transmitted via a Bluetooth connection at a rate of one sample per minute to a smartphone running the Empatica Care Lab application. This application was used to transfer the data to an FDA-compliant online repository. To maintain data integrity and reliability, a manual inspection process was performed to identify and correct potential errors or inconsistencies. For the evaluation, the datasets were analyzed using Python version 3.9.13, Pandas version 2.2.1, and scipy version 1.13.1.

## 4. Design and Implementation

The two systems utilized for the study are fundamentally based on the same concept of implementing sensor-assisted training on the ICAROS Pro. The system evaluated in the main study had previously undergone testing in [16], whereas the system evaluated in the secondary study is a self-developed exergame.

### 4.1. Main Study of Training System

The main study was based on a feasibility study [16] to evaluate the training system and training progress. After consultation with the sports manager and fitness trainer involved, the previously examined rehabilitation-focused procedure was modified to a strenuous training procedure. The reason for this paradigm change was that the ICAROS Pro is primarily designed to be operated by physically fit persons.

#### 4.1.1. System Architecture

**Figure 6:**
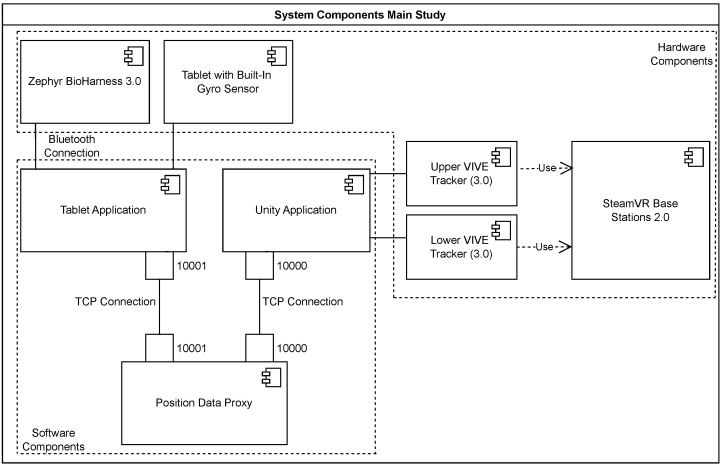
Main study system architecture, showing data flows and component interactions.

This section explains how the components work together during the training process as shown in Figure 6. The two tracking sensors attached to the back of the subject provide the posture and rotation data to the computer. The computer reads position and rotation data using the Steam VR plugin and the Unity 3D engine, transmitting these to the tablet attached to the ICAROS Pro via a Transmission Control Protocol (TCP) connection. The tablet also has a Bluetooth connection to the Zephyr BioHarness 3.0, which transmits heart rate and respiration rate using the Zephyr protocol. The application also uses the gyro sensor of the tablet to determine pose. The data produced by the application is stored in a SQLite database. The exact structure of the database is shown in [16]. It is not possible to access the data from outside the application. A user must be logged in to be able to export their data as a Comma Separated Values (CSV) file.

#### 4.1.2. Tablet Application

**Figure 7:**
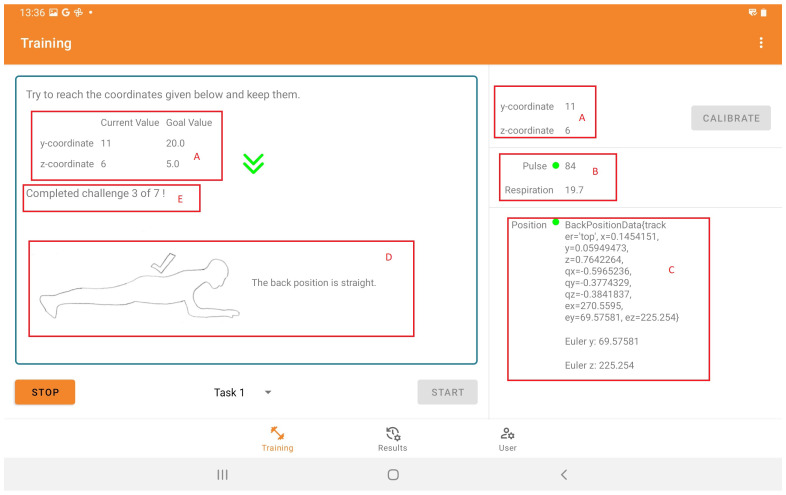
Tablet application screenshot: The app displays data from the gyro sensor (A), the connected Zephyr BioHarness 3.0 (B), and Vive Tracker position/alignment (C). Guidance shows the current and the target position (A), the current back posture (D), and session progress (E).

Initially, users create a password-protected account via the tablet application and enter their information, e.g., weight, sex, height, and date of birth. It is also possible to set up the current status of the connection to the TCP proxy within the application. In Figure 7, a view of the training app is shown. In the top right of the screen, the current connection to and values of the Zephyr BioHarness 3.0, the connection to the proxy, and the position and orientation of the trackers are displayed. The current status of the training is displayed on the left side of the screen. In addition to the current position of the user, the target position is shown. The posture of the user is also shown. The developed app was programmed bilingually in German and English to allow a broader user group.

#### 4.1.3. Training Procedure

In consultation with a sports manager and fitness trainer, the training procedure was developed based on the specific plank position exercises of the ICAROS Pro with back posture tracking. The training procedure consisted of three training sessions per subject, including three tasks. Each task was limited to a duration of 52 s, divided into seven pose goals with a certain holding time (see Table 4). These pose goals are defined by certain angles for each ICAROS Pro axis. Goal 1 represents a fixed starting pose, while goals 2/3, 4/5, and 6/7 represent pairs with inverted angles on the X/Roll axis. The order of tasks was altered during each training session; pose goals pair orders and succession were switched systematically as well (see Table 5). This was applied to avoid a potential bias caused by muscle memory. Training sessions had to be performed at least two days apart, and a break of two minutes was maintained between tasks. For each training session, subjects were able to choose dates within a specified time frame, ranging from 9:30 am to 3:00 pm. To ensure comparability, subjects were encouraged to reserve the same time slots as possible. The provision of flexible scheduling and the option to cancel were designed to minimize stress. The main observation period was two months.

Before starting each training session, the subjects received an introductory explanation of the study, the functioning of the training system, and the training procedure. Before starting the exercise, subjects were asked whether they felt comfortable and ready to proceed, followed by registration or login to the tablet application. The training equipment was adapted to the body measurements of the subjects. Subjects had the option of wearing the Zephyr BioHarness 3.0 due to direct skin contact. After an adaptation phase on the ICAROS Pro, the starting position of the back was calibrated to (0, 0) by aligning the VIVE Tracker (3.0) to calculate back posture. Using Euler angle differences between the start position and current position during training, the back posture is recorded. A tolerance of ±3° is integrated. Exceeding the limit, the incorrect back posture is estimated and signaled to the subject. After calibrating, the training session is started.

#### 4.1.4. Training Setup

**Figure 8:**
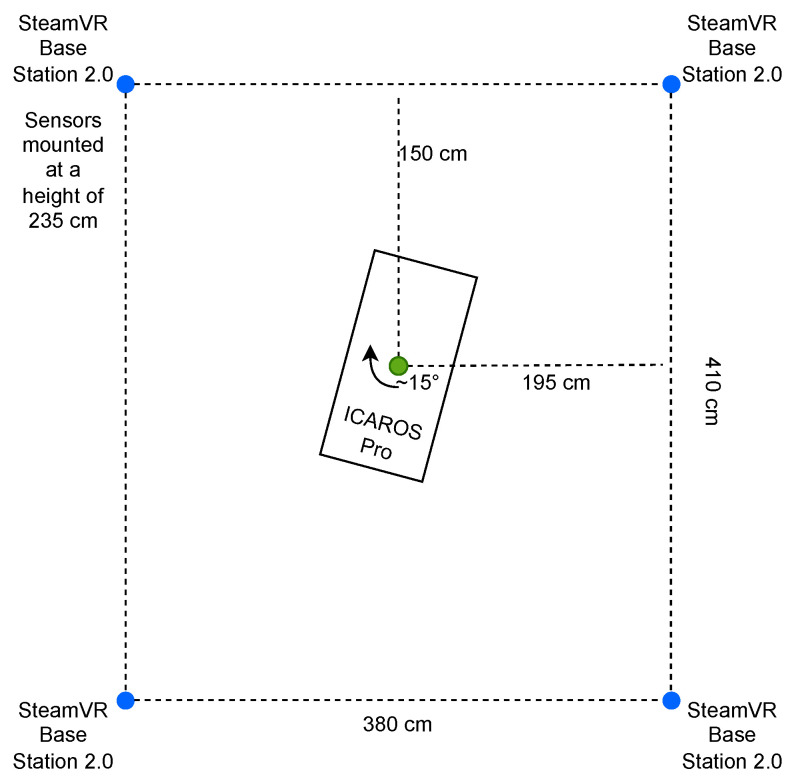
Positioning of components within experimental setups: The ICAROS Pro was placed mildly tilted among four SteamVR Base Station 2.0, positioned in the corners of a rectangular area.

The ICAROS Pro is located in the center of the area, covered by the SteamVR Base Stations 2.0, as shown in Figure 8. The device was rotated 15° clockwise and faced the center of the room. The point marked within the ICAROS Pro represents the pivot point of its axes. The SteamVR Base Stations 2.0 are placed at a height of 235 cm. Base Stations 2.0 on the right were mounted on a wall, and the ones on the left on tripods.

### 4.2. Secondary Study of VR-Based Exergame

This study used a self-developed VR-based exergame controlled by the pose of the player on the ICAROS Pro while wearing an HMD. The goal of the game was to collect virus particles with the help of a drone while flying through the bloodstream of a vessel. The same HTC VIVE Trackers (3.0) used in the main study were used to control the game, now attached to the ankles of a player. However, the participating subjects wore the EmbracePlus as an alternative to the Zephyr BioHarness 3.0 for pulse rate recording.

#### 4.2.1. System Architecture

**Figure 9:**
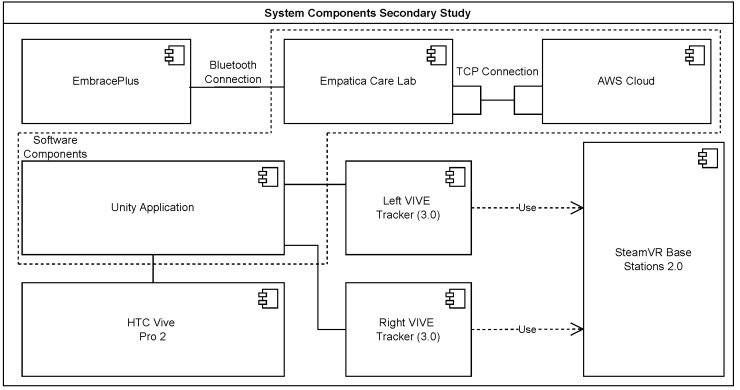
System architecture of the secondary study, showing the data flows and interactions between the different system components.

This section describes the components of the VR-based exergame study as shown in Figure 9. As in the main study, the two sensors are attached to the subjects. Instead of being attached to the upper and lower back, the sensors are attached to the left and right ankle. The PC connected to the sensors now uses SteamVR and the Unity3D game engine to capture the Y-coordinates of the two sensors. The transmission of the vital parameters via the EmbracePlus was realized with the help of a smartphone on which the Empatica Care Lab is installed, using an encrypted Bluetooth connection. This application was used to transmit the data to an FDA-compliant online storage.

#### 4.2.2. VR Interface

**Figure 10:**
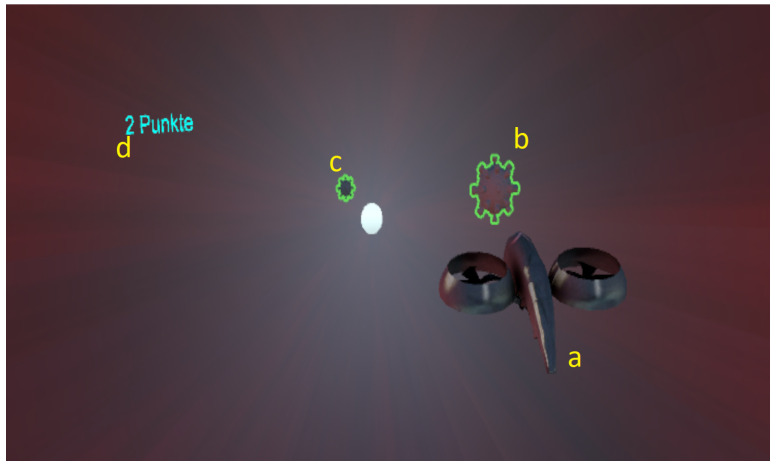
Screenshot of the VR exergame interface: In a third person view, a drone (a) is steered by position adjustments of the player. The steadily moving drone is to be guided to target a continuously emerging series of virus particles (b, c). Successful “catches” are counted as game score (d).

As soon as the game is started, the player will see a screen with prompts for the calibration positions to be recorded. Once all positions are recorded, the drone moves forward at a constant speed. The player can now control the position of the drone by moving his body on the ICAROS Pro as can be seen in Figure 10. The locations of the viruses to be captured by the player are static for the first four viruses and are randomly generated starting with the fifth virus. The distance and therefore the time between each virus is the same. Each time the drone flies through a virus, a sound is emitted via the HMD and the score is increased by one point. The score starts at 0 points. After the player has flown past or collected 30 viruses, the game ends and the final score is displayed.

#### 4.2.3. Training Procedure

Before the first training session, the subjects were given the opportunity to test the ICAROS Pro to feel comfortable wearing the VR headset while positioned on the ICAROS Pro. Furthermore, the procedure of the game was explained to the subjects. At the beginning of each training session, the resting heart rate was measured for several minutes using the EmbracePlus. After this rest phase, the exergame was calibrated. At the beginning of each session, users can set positions on the ICAROS Pro that they can reach during the calibration phase as the outermost positions for the respective game session, which determine the playing field in which they can later move. During this calibration phase, the coordinates are recorded for the following five positions: (i) rear position, (ii) center position, (iii) front position, (iv) left tilt position, and (v) right tilted position.

Only the Y coordinates are recorded by the sensors during the calibration phase and during the gameplay. To determine the height of the player in the game, the sum of the Y coordinates is calculated and compared with the values recorded during calibration. The exact position is determined by interpolating the sum. The calculation for the position on the X axis is similar, except that the difference is calculated instead of the sum of the coordinates. If the difference between the two coordinates is negative, the player is tilted to the left; if the difference is positive, the player is tilted to the right. Again, the exact position is interpolated using the current values and the values recorded in steps (iv) and (v). The next step of the training was the 3-min exercise portion of the training, followed by five to ten minutes of recovery. This time was used to determine how long it would take the subject to return to their previous resting heart rate. At the end of the third study, subjects were asked to complete a survey to evaluate the training.

#### 4.2.4. Training Setup

Unlike the main study, the secondary study did not use the tablet mounted on the ICAROS Pro. Instead, the player used the HTC VIVE Pro 2. In addition, vital signs were not recorded using the Zephyr BioHarness 3.0, but via the Empatica EmbracePlus worn on the wrist. Another person is also required to calibrate the game. The remaining structure of the study is identical to the structure shown in Figure 8.

## 5. Results

This section analyzes and describes the data obtained in the relevant studies. In addition to the recorded vital signs, this includes the results of the training systems.

### 5.1. Main Study of Training System

This section presents an evaluation of the study using three primary specific parameters: Back Posture, Heart Rate, and Respiration Rate. Of the initial 22 subjects, 20 completed all three training sessions. During these sessions, every subject was equipped with the Zephyr BioHarness 3.0 monitored the pulse and respiration rates. All 20 subjects responded to the retrospective question regarding their fitness at the time of participation. The recorded measurements were carefully reviewed for potential errors, and any identified errors were manually excluded. The filtered data were then analyzed to determine the statistical key figures for each parameter. After an initial descriptive data analysis, the dataset was examined for correlations between the different variables. This analysis revealed striking correlations between heart rate and level of fitness, respiration and BMI, incorrect back posture and BMI, and respiration and sex. Because some of the correlations were conspicuous, the effects of the variables level of fitness, BMI, and sex on the variables heartbeat, respiration, and incorrect back postures were further examined.

#### 5.1.1. Heart Rate

Table 6 shows the statistical key figures of the heart rates of the subjects subdivided into the different training sessions. During the first training session, the subjects showed an average heart rate of 90±10 bpm [median: 89.50 bpm; 95% CI: 87.33,92.87], with a maximum recorded value of 112 bpm and a minimum of 66 bpm. In the second training session, the average heart rate decreased slightly to 87±12 bpm [median: 87.69 bpm; 95% CI: 84.49,90.88], although the maximum heart rate increased to 123 bpm, while the minimum remained consistent at 66 bpm. In the final training session, the average heart rate decreased further to 86±10 bpm [median: 85.50 bpm; 95% CI: 83.47,88.53]. Specifically, the minimum heart rate increased to 70 bpm, while the maximum heart rate reached its lowest value of the sessions at 104 bpm. The decreasing trend in the average heart rate across the individual sessions is particularly notable. This development could be an indication that the subjects have started to get used to the type of training.

**Figure 11:**
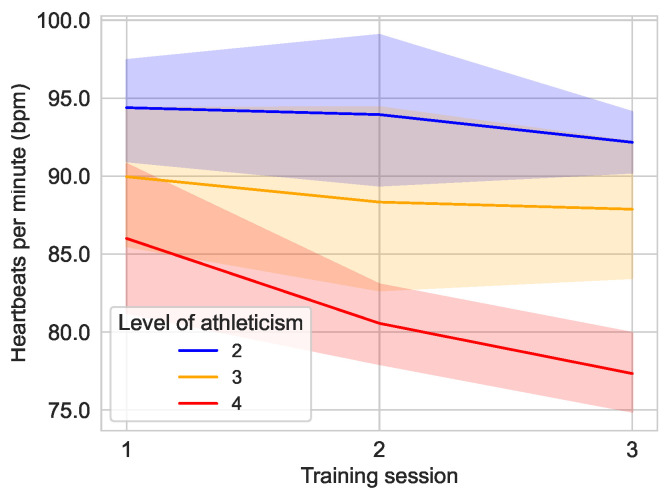
Average heart rate over the course of the different training sessions in the main study divided by the fitness level of the user. No users classified themselves as unfit (1) or fit (5), and the remaining groups with fitness level of 2 (n=6), 3 (n=8), and 4 (n=6) are shown in their designated color.

A breakdown of the study population based on the self-reported fitness of the subjects shows a difference in the observed heart rate in Figure 11. For example, the group of subjects who rated their fitness as rather unfit (2) (n=6) had an average heart rate of 94.39±7.55 bpm [median: 96.00 bpm; 95% CI: 90.90,97.88] in the first training session, while it was 93.94±10.79 bpm [median: 93.00 bpm; 95% CI: 88.96,98.93] in the second training session and fell to 92.16±4.41 bpm [median: 92.00 bpm; 95% CI: 90.13,94.20] in the third training session. The group of moderately fit (3) subjects (n=8) had an average heart rate of 87.67±11.13 bpm [median: 89.00 bpm; 95% CI: 85.08,94.83] in the first session, 88.57±16.17 bpm [median: 86.50 bpm; 95% CI: 82.29,94.38] in the second session and 88.52±11.78 bpm [median: 83.50 bpm; 95% CI: 83.39,92.36] in the last session. The group of rather fit (4) subjects (n=6) had a heart rate of 86.00±10.94 bpm [median: 84.00 bpm; 95% CI: 80.94,91.06] at the first training session. The heart rate of the group decreased to 80.56±5.70 bpm [median: 80.00 bpm; 95% CI: 77.93,83.18] in the second session and decreased further to 77.33±5.76 [median: 75.50 bpm; 95% CI: 74.67,79.99] in the third session.

**Figure 12:**
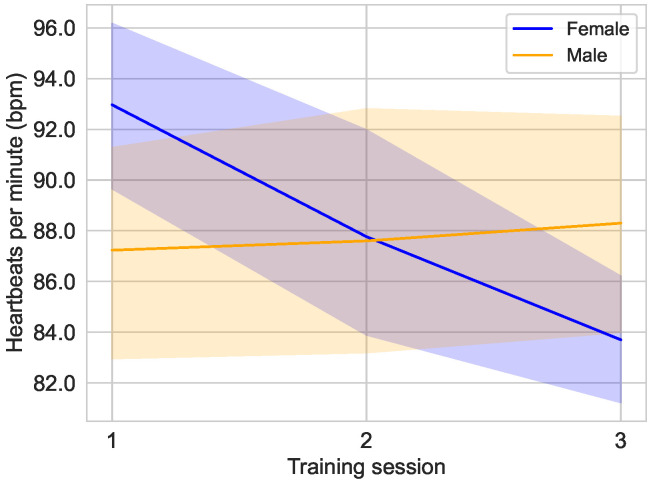
Average heart rate over the course of the different training sessions in the main study divided by the sex of the user, female (n=10) and male (n=10).

A sex-related difference in the heartbeat rate progression has been identified, as can be seen in Figure 12. There was little change in average pulse rate in the male group with 87.23±11.54 bpm [median: 88.00 bpm; 95% CI: 83.11,91.36] in the first training session, 87.60±13.69 bpm [median: 85.00 bpm; 95% CI: 82.70,92.50] in the second, and 88.30±11.87 bpm [median: 90.00 bpm; 95% CI: 84.05,92.55] in the third. In the female group, it decreased considerably, with 92.97±9.65 bpm [median: 94.00 bpm; 95% CI: 89.51,96.42] in the first training session, 87.77±11.71 bpm [median: 86.00 bpm; 95% CI: 83.58,91.96] in the second, and 83.70±7.21 bpm [median: 83.50 bpm; 95% CI: 81.12,86.28] in the third.

**Figure 13:**
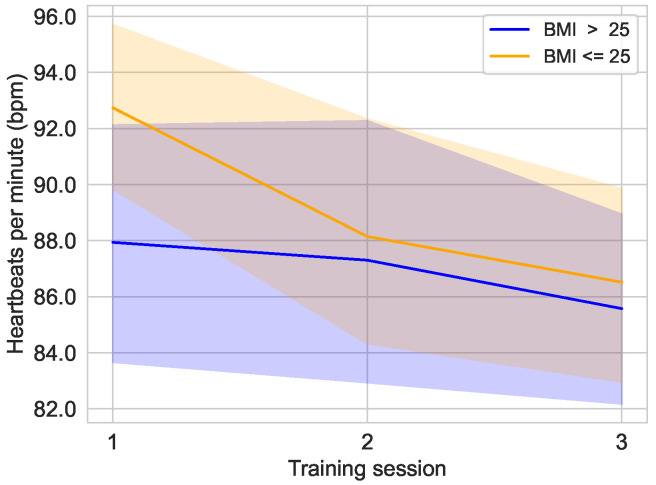
Average heart rate over the course of the different training sessions in the main study divided into the groups BMI≤25 (n=9) and BMI>25 (n=11).

Figure 13 shows the progression of heart rate during the three training sessions, divided into groups based on the threshold of BMI≤25 and BMI>25. It is clear that after an initial difference in the first training session, the heart rates had equalized and from the second training session were only 0.8 bpm to 0.9 bpm apart. Overall, there was a slight downward trend in both groups. The group with a BMI≤25 had an average value of 92.74±8.17 bpm [median: 93.00 bpm; 95% CI: 89.66,95.82] in the first training session, 88.15±10.34 bpm [median: 86.00 bpm; 95% CI: 84.25,92.05] in the second training session, and 86.52±9.65 bpm [median: 87.00 bpm; 95% CI: 82.88,90.16] in the last training session. Meanwhile, the BMI>25 group had an average heart rate of 87.94±12.47 bpm [median: 88.00 bpm; 95% CI: 83.68,92.19] in the first session, 87.30±14.38 bpm [median: 85.00 bpm; 95% CI: 82.40,92.21] in the second session, and 85.58±10.42 bpm [median: 85.00 bpm; 95% CI: 82.02,89.13] in the last session. The average BMI of the female group was 25.11 and it was 26.29 in the male group.

#### 5.1.2. Respiration Rate

Table 7 shows the statistical key figures of the total group divided into the three different training periods. A clear change in respiration rate is not visible, but in addition to a reduction in the maximum respiration rate, a reduction in the standard deviation can be observed.

**Figure 14:**
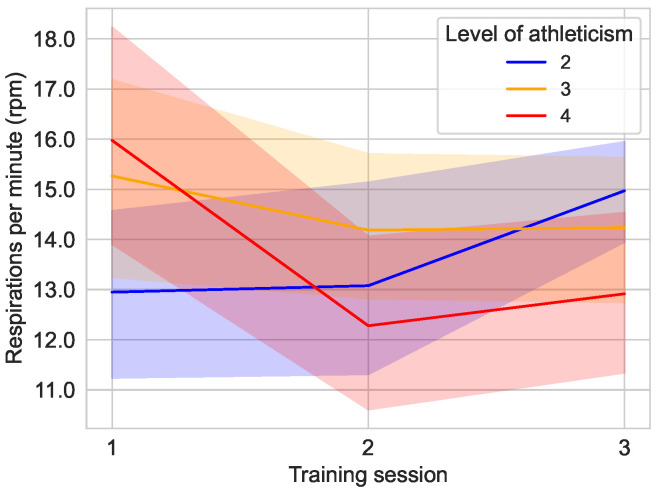
Average respiration rate over the course of the different training sessions in the main study divided by the fitness level of the user. No users classified as unfit (1) or fit (5), and the remaining groups with fitness level of 2 (n=6), 3 (n=8), and 4 (n=6) are shown in their designated color.

The development of the respiration rate according to the self-assessed fitness level does not show a clearly recognizable pattern, as can be seen in Figure 14. For example, the respiration rate of users with fitness level 2 increases from the second training session, while the fitness level 4 group initially shows a sharp decrease and then a slight increase in the third training session.

**Figure 15:**
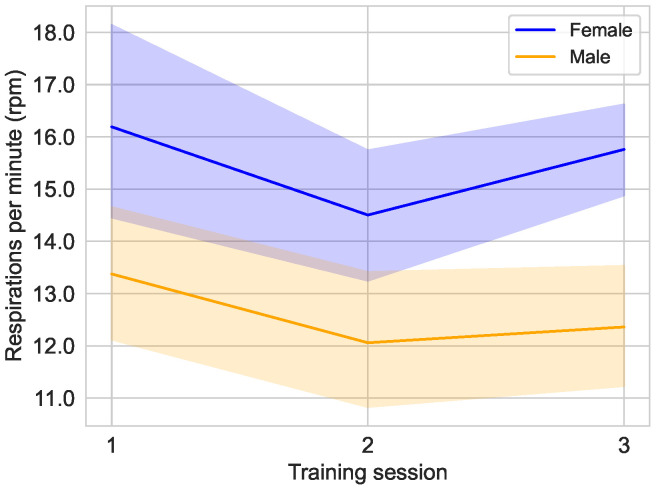
Average respiration rate over the course of the different training sessions in the main study divided by the sex of the user, female (n=10) and male (n=10).

As can be seen in Figure 15, the average respiration rate of the male subjects in the study is considerably lower than that of the female subjects. In both groups, in addition to a decrease in respiration rate in the second training session, there is an increase in respiration rate in the third training session. However, this increase in the third training session is much greater in the female group than in the male group. For the male subjects, the respiration rate decreases from 13.38±3.55 rpm [median: 13.00 rpm; 95% CI: 12.10,14.65] in the first training session to 12.06±3.83 rpm [median: 12.01 rpm; 95% CI: 10.69,13.43] in the second training session and increases slightly to 12.36±3.29 rpm [median: 12.90 rpm; 95% CI: 11.18,13.54] in the third training session. For the female subjects, the respiration rate first decreases from 16.19±5.40 rpm [median: 15.54 rpm; 95% CI: 14.26,18.13] to 14.60±3.65 rpm [median: 14.43 rpm; 95% CI: 13.20,15.81] and increases to 15.76±2.39 rpm [median: 16.18 rpm; 95% CI: 14.90,16.61] in the last training session.

**Figure 16:**
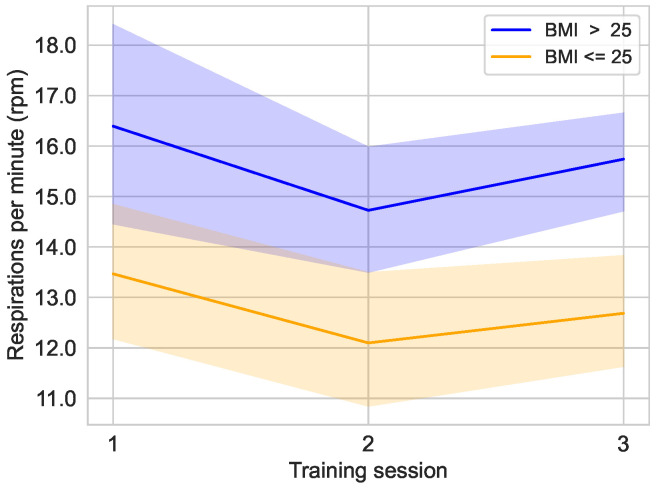
Distribution of respiration rates over the training sessions in the main study divided into the groups BMI≤25 (n=9) and BMI>25 (n=11).

Figure 16 shows the course of the respiration rate over the three training sessions, divided into groups based on the threshold of BMI≤25 and BMI>25. It is obvious that the average respiration rate of the group of users with a BMI>25 is clearly lower than that of the group with a BMI≤25. The respiration rate of the BMI≤25 group was 16.39±5.21 rpm [median: 15.13 rpm; 95% CI: 14.43,18.36] in the first session, 14.73±3.34 rpm [median: 14.40 rpm; 95% CI: 13.47,15.98] in the second session, and 15.74±2.66 rpm [median: 16.38 rpm; 95% CI: 14.74,16.74] in the last session. Meanwhile, the BMI>25 group had respiration rates of 13.47±3.95 rpm [median: 13.39 rpm; 95% CI: 12.12,14.82] in the first, 12.10±3.99 rpm [median: 11.68 rpm; 95% CI: 10.74,13.46] in the second, and 12.69±3.21 rpm [median: 13.07 rpm; 95% CI: 11.59,13.78] in the last session.

#### 5.1.3. Back Posture

Analysis of the number of incorrect back postures is used to determine the effectiveness of the training. As shown in Table 8 the average number of incorrect back postures goes from 13.87±11.57 [median: 11.00; 95% CI: 10.94,16.79] incorrect postures in the first training session to its peak value of 15.10±11.19 [median: 13.00; 95% CI: 12.27,17.93] postures in the second training session to a final value of 12.95±9.51 [median: 11.00; 95% CI: 10.54,15.36] incorrect back postures in the third training session. The minimum value of the incorrect back postures in the first and second training sessions was 0 incorrect back postures, and it went up to one incorrect back posture in the final training. The maximum number of incorrect back postures went down from 54 incorrect back postures in the first training to 39 incorrect back postures in the second training to 36 incorrect back postures in the last training session.

**Figure 17:**
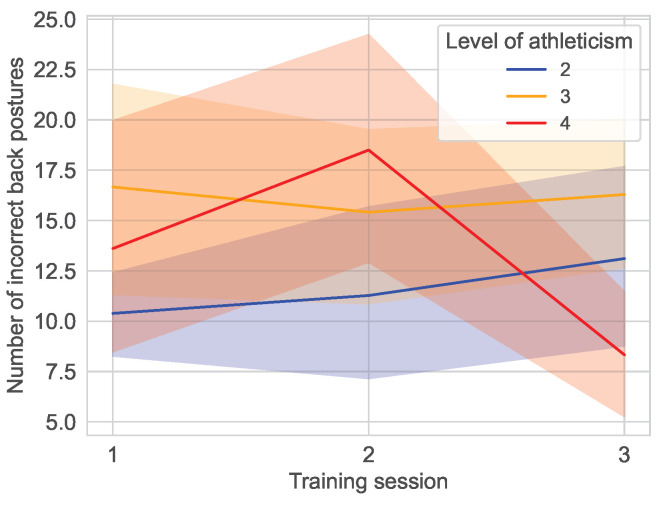
Distribution of incorrect back posture over the training sessions in the main study divided by the fitness level of the user. No users classified themselves as unfit (1) or fit (5), the remaining groups with fitness level of 2 (n=6), 3 (n=8) and 4 (n=6) are shown in their designated color.

Figure 17 presents the progression of the average incorrect back positions, categorized by levels of fitness. Group 4 (the “fit” group) demonstrates a notably divergent progression in contrast to the other two groups. The “fit” group exhibits considerable fluctuations in the number of errors, suggesting potential variability in their training or performance. The initial mean ± standard deviation of the group for incorrect back postures was 13.61±12.60 [median: 11.00; 95% CI: 7.80,19.42], which increased to 18.50±12.15 [median: 21.00; 95% CI: 12.89,24.11] in the second training session and decreased to 8.33±6.94 [median: 6.00; 95% CI: 5.13,11.54] in the last training session.

**Figure 18:**
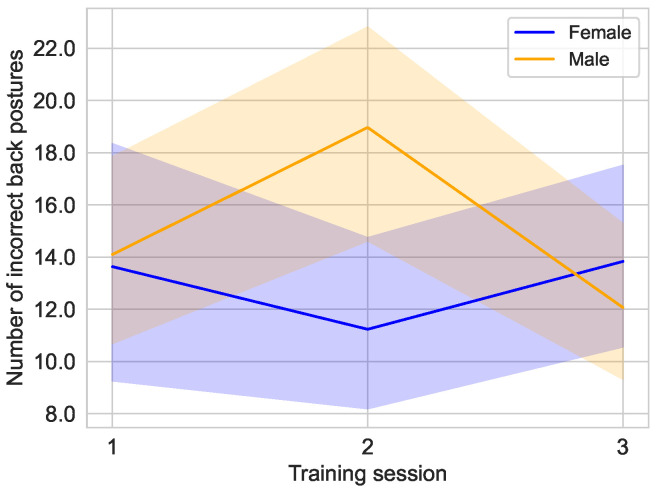
Distribution of incorrect back posture over the training sessions in the main study divided by the sex of the user, female (n=10) and male (n=10).

As Figure 18 shows, the average number of incorrect back postures in the male subjects initially increases after the first training session from 14.10±9.96 [median: 12.50; 95% CI: 10.54,17.66] to 18.97±11.66 [median: 21.50; 95% CI: 14.80,23.14] in the second training, but decreases to its lowest value in this group of 12.07±8.89 [median: 9.50; 95% CI: 8.88,15.25] in the last training session. An opposite effect can be seen for the female subjects. After the average number of incorrect back postures in the first training session is almost identical to that of the other group (13.63±13.16) [median: 9.00; 95% CI: 8.93,18.34], it drops to 11.23±9.37 [median: 9.50; 95% CI: 7.88,14.59] in the second training session and rises to the maximum value for this group of 13.83±10.16 [median: 11.50; 95% CI: 10.20,17.47] in the last training session.

**Figure 19:**
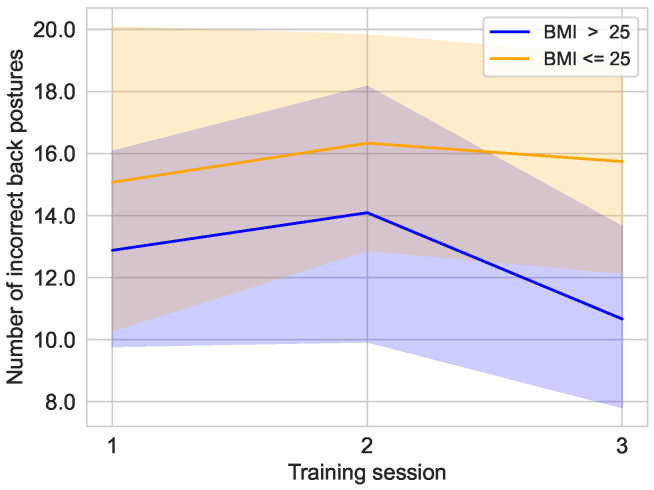
Distribution of incorrect back posture over the training sessions in the main study divided into the groups with a BMI≤25 (n=9) and a BMI>25 (n=11).

As Figure 19 shows, the BMI>25 group was able to achieve a lower number of incorrect back positions over the training sessions. In the first training session, the average number of incorrect back posture in the BMI>25 group was 12.88±9.87 [median: 11.00; 95% CI: 9.51,16.25], compared to 15.07±13.46 [median: 11.00; 95% CI: 10.00,20.15] in the BMI≤25 group. The second training showed an average number of incorrect back positions for the BMI>25 group of 14.09±12.57 [median: 10.00; 95% CI: 9.80,18.38], compared to 16.33±9.32 [median: 16.00; 95% CI: 12.82,19.85] for the BMI≤25 group. The gap between the groups widened in the third training, where the BMI>25 group averaged only 10.68±9.11 [median: 9.00; 95% CI: 7.56;13.78] incorrect postures, compared to 15.74±9.39 [median: 13.00; 95% CI: 12.20,19.28] incorrect postures for the BMI≤25 group.

### 5.2. Secondary Study of VR-Based Exergame

In addition to the parameters recorded by the Empatica EmbracePlus, such as heart rate and EDA value, the points achieved during the game and the results of the survey are evaluated in this section. During the training sessions, the recorded heartbeat rate was checked for errors using the Care Lab app, which resulted in one of the subjects changing the wrist on which the EmbracePlus was worn. The recorded data were manually checked for errors during analysis. This review revealed some errors within the recorded EDA values. As a result, only segments of the EDA data were used for visualization.

#### 5.2.1. Heart Rate

**Figure 20:**
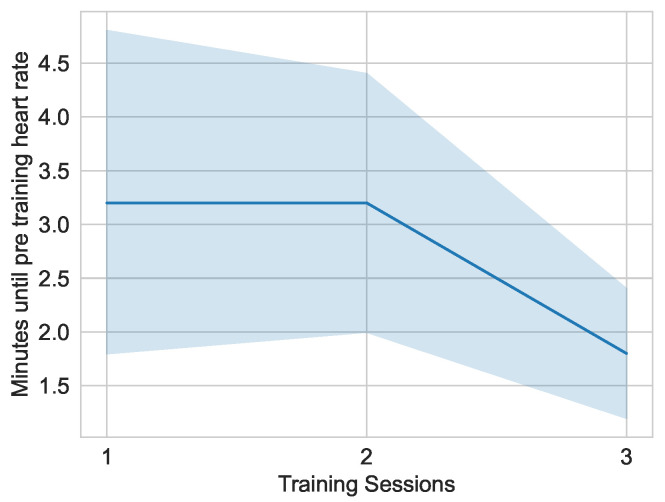
Distribution of time until subjects reach their pre-training heart-rate, divided into the different training sessions in the secondary study (n=5).

Figure 20 shows the time required for the subjects to return to their pre-training heart rate after completing the game. The time required for the first and second training sessions was on average 3.2 min. What is striking about the graph is the reduction in the time required in the last training session with an average of 1.8 minutes. It is also notable that the deviation from the average time decreases as the training progresses: (1.79, 1.48, 0.84).

#### 5.2.2. Game Score

Table 9 shows the statistical figures for the archived score divided into the separate training sessions. As the maximum number of points achievable is 30, the average number of points achieved by subjects was very high. Looking at Table 9, the increased standard deviation in the second training session is noticeable. This is due to a considerably lower score for one subject in the second training session. After the training session, the subject noticed that their position on the ICAROS Pro was different than during the first training session.

#### 5.2.3. Survey Results

**Figure 21:**
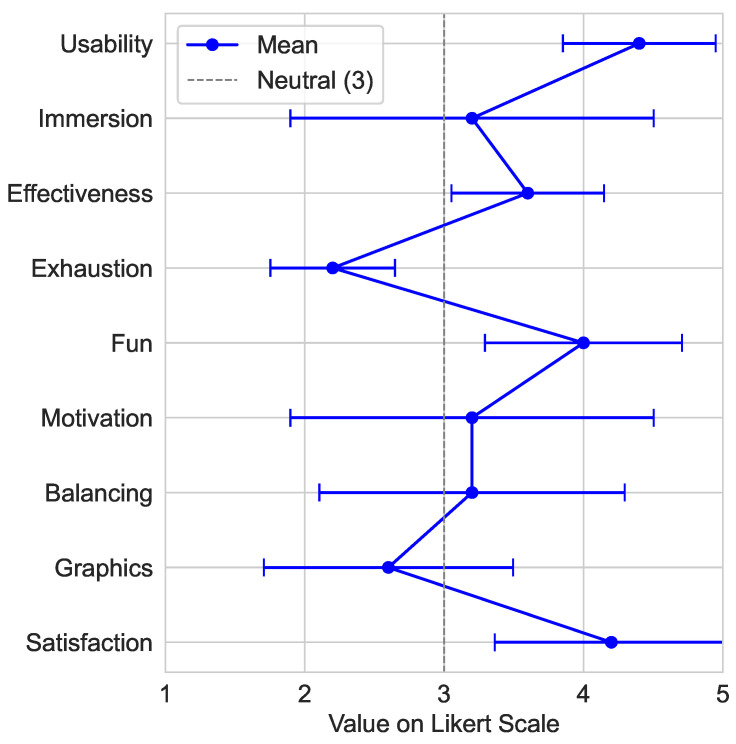
Profile line of mean values with standard deviations for evaluation questions of the Likert type (1–5, Low/Poor–High/Good).

The survey results are elaborated in this section, and the results of the Likert type questions are additionally visualized in Figure 21.

The aspect Usability was rated rather easy up to easy to use (4.4±0.55). The Immersion was rated moderate (3.2±1.30). In regard to Adversities, none were reported. The aspect Effectiveness of the training was rated moderately up to rather effective (3.6±0.55). All subjects reported the back (n=5) as the main muscle group trained, but some also reported arms (n=2), legs (n=1), abdomen (n=1), chest (n=0), and neck (n=1). However, Exhaustion was rated rather not strenuous (2.2±0.45). The aspect Fun of the game was rated rather fun (4.0±0.70). Motivation was rated as moderately motivating (3.2±1.30). The aspect Balancing between challenge and reward was rated as moderately balanced (3.2±1.10). The Graphics were rated rather poor up to moderately qualitative (2.6±0.89). In regard to Bugs, one subject reported a problem. The scene appeared distorted, resulting in poor readability of text elements. Satisfaction was rated as rather satisfying (4.2±1.14). In regard to Recommendation, all subjects (n=5) would recommend the game to others. One subject used the question Feedback to explicitly mention that the game was fun.

## 6. Discussion

In the following sections, the results of both studies are discussed, and the strengths and weaknesses of each are identified. Further research opportunities are then suggested.

### 6.1. Main Study of Training System

The findings of the preliminary study demonstrate a positive correlation between heartbeat rate and self-assessed fitness level, supporting the hypothesis that the study subjects were able to accurately categorize their level of fitness. In addition, Figure 11 reveals a substantial decrease in the average heartbeat rate in the most fit group (level of fitness 4) over the course of training. This group demonstrates the greatest adaptability to the physical demands of training. A notable observation in Figure 12 is the decrease in heart rate among female subjects during the training period, while male subjects exhibited a slight increase in heart rate. This finding suggests a potential adaptation difference between the sexes, with female subjects demonstrating a more pronounced ability to adapt to the training regimen. This finding is particularly noteworthy given that the female subjects reported a lower average level of fitness (2.8) compared to the male subjects (3.2).

The findings of the respiration rate analysis demonstrate a correlation between the sex of the subjects and the respiration rate, as well as between the BMI of the subjects and the respiration rate. As illustrated in Figure 15, female subjects exhibit a conspicuously higher respiration rate compared to the male group. A comparison of the figures in Figure 15 and Figure 16 reveals a discernible similarity between the influence of the variables BMI≤25 and sex. This similarity in the influence of these variables may be primarily attributable to the distribution of BMI across the two sexes, which is 25.11 for female subjects and 27.46 for male subjects (see Table 1).

The analysis showed a slightly lower number of incorrect back postures in subjects with a BMI>25, as can be seen in Figure 19. This difference may be due to the attachment of the motion trackers to the subjects. The straps used had a rather small contact area to which a tracker was mounted, allowing for mild movement. Such movements may have been dampened in subjects with more muscle mass or a thicker layer of fat on their backs. Subjects with lower BMI reported hearing the error tone more frequently, despite maintaining “the same back posture”.

A strength of the conducted study lies in the representativeness and safety of the training procedure. The sports manager consulted highlighted that training sequences longer than 1 min may overwork subjects and may even have adverse effects when holding a posture with a sagging spine for too long. Audio-visual feedback during training informed subjects about such risks. In addition, lab personnel monitoring the training sessions was attentive to potentially intervene, even though no adverse situation occurred. Sequences of pose goals and tasks within training sessions were further designed to minimize bias by shuffling and inverting pose goal pair orders, thus potentially induced by muscle memory.

One limitation of the system is the fixed parameters, e.g., the tolerance for calculating incorrect back positions can only be adjusted by changing the source code. Another weakness of the study is that the group of subjects was very homogeneous, mainly students in their mid-twenties to mid-thirties. Two subjects did not complete the study and the reasons for their withdrawal were not specified. A rather frequently observed issue during training sessions was related to the gyro sensor of the tablet, causing mild to severe rotational drifts. Involved personnel actively tracked occurrences and severity of drifts, and re-started affected sessions in case they exceeded mild severity. Therefore, in cases where only a minimal change in position was required, subjects may have performed slightly adapted positions while adhering closely to the training plan. The continuous monitoring minimized gyro drift, reducing its potential impact on the results. The cause of drifts is suspected to be that the tablet used in the mount on the ICAROS Pro was exposed to slight vibrations that caused this drift. A potential solution to this issue involves the utilization of an alternative mount for the tablet. The implementation of a new-generation tablet with upgraded and more robust gyro sensors could also solve the problem. Alternatively, the implementation of a different system for determining the position of the ICAROS Pro, similar to the approach employed in the secondary study with the VR hardware, could be considered.

One issue that occurred relates to the heartbeat rate. In four cases, the recorded heartbeat rate was 0 bpm. In three of the four cases, the values marked with 0 were removed from the dataset. In the fourth case, when checking the data, in addition to the incorrect recording, values lower than the normal heartbeat rate for this subject were also recorded in all three training components in the first training session. In this case, the first training session was repeated by the subject several months later. This problem caused parts of the respective training sessions to be missed by the subject. The repetition of the first training of the subject could have an influence on the results due to other environmental influences and a possible habituation of the subject to the training. To prevent this, the repetition was only carried out after several months and the subject was asked if their lifestyle had changed since the rest of the training, which the subject denied. The reason for the incorrect data collection is assumed to be that the minimum setting of the Zephyr BioHarness 3.0 is too large, as the majority of the subjects have a relatively small chest circumference. One way of preventing this error is to livetrack the data, which issues a warning if the recorded heartbeat rate is 0 bpm while the connection to the Zephyr BioHarness 3.0 is active. However, it is noteworthy that the respiration rate continued to be recorded while the heart rate was not recorded.

In regard to the generalization of results, it should be considered that the subjects of the study were primarily young individuals. Due to these demographic limitations as well as the rather small study population, results may not be generalized to the broader population, particularly with respect to older adults or individuals with varying levels of physical fitness.

### 6.2. Secondary Study of VR-Based Exergame

During the secondary study, technical problems with the system were identified at one point by the test subjects and the supervisor. This problem consisted of the poor legibility of the text during the calibration phase of the game. This problem was only reported by the subject during one of the three training runs, so this problem was most likely caused by an incorrect setup of the HTC VIVE Pro 2 before the start of the training. Apart from this problem, the feedback from the test subjects was predominantly positive, as can be seen in Figure 21. The usability with a mean of 4.4±0.55 and the fun factor, averaging 4.0±0.71, received particularly favorable ratings. The training effectiveness rating on average by 3.6±0.55 was above average, and the perceived effort level was rated as less exhausting than traditional training with a mean of 2.2±0.45. Beyond the survey answers, all subjects offered additional feedback on the developed system, mentioning its enjoyable nature. Furthermore, there was a recurring theme in the feedback regarding the preference for a more diverse gaming experience, exemplified by the suggestion of different levels of difficulty and game modes, such as obstacle avoidance or a restricted field of vision. The majority of subjects (n=4) had previous experience with VR systems. This could have affected achieved scores for certain aspects in a positive (e.g., Usability, Satisfaction) or a negative (e.g., Immersion, Graphics) way. A study group involving solely inexperienced subjects might thus have judged notably different in regard to certain aspects. Those familiar with VR could adapt more easily, while inexperienced users faced greater difficulty due to the combination of visual impairment and the unfamiliar ICAROS Pro device. This initial disorientation could lead to reduced enjoyment among subjects unfamiliar with VR.

The EmbracePlus data collection process revealed issues with the heartbeat rate monitoring during one session. During the pre-training phase, the Care Lab app monitored the heartbeat rate of the subjects and recorded an unusually low value using the EmbracePlus. Upon noticing this, the fitting of the smartwatch was adjusted. When this was noticed, the fit of the smartwatch was adjusted, but this did not lead to a correct recording. Changing the wrist on which the EmbracePlus was worn then led to a correct recording of the heartbeat rate. However, a notable limitation of the EmbracePlus system is its inability to record heart rate data more frequently than once per minute, a limitation that is further compounded by the relatively brief duration of the individual training sessions. An analysis of the recorded data revealed that the EDA values were not accurately recorded in four out of five subjects. In multiple instances, the recordings indicated that the EDA values remained constant throughout the training period at a very low level. Additionally, there was one subject with a much higher EDA value than the rest of the subjects. Some individuals also exhibited higher values in the pre-training phase compared to the training phase. One potential explanation for this phenomenon, in addition to the possibility of inaccurate data recording, could be the occurrence of fluctuating temperatures during the training sessions, since the exercises were not performed under laboratory conditions for specific training sessions. On several occasions, the outside temperatures at the time of the training sessions exceeded 30 °C. This could have an impact on the initial observation. Based on this, the EDA score was not considered further in the analysis. Moreover, the data recording was reduced for certain test subjects who arrived later than scheduled for the training sessions. Consequently, the pre-training phase was shortened to extend the duration of the post-training recordings. All subjects were required to wear the EmbracePlus until the recorded heartbeat rate reached the pre-training value.

### 6.3. Future Work

Further development of the exergame would be possible by expanding the game modes. Two possible game modes suggested by the testers were a restricted field of vision for the player or adjusting the flight speed to the current heart rate of the player. Furthermore, an endless mode would be possible in which the player succeeds until a predetermined number of viruses are missed. A combination of the two systems would also be possible. In this way, the playful approach of the exergame, including the visualization of the information on the HMD, could be combined with the back tracking of the training system. One option would be to only award points to the player if their back posture is correct. It would also be possible to equip the ICAROS Pro with pressure sensors on the contact surface of the knees and arms to prevent incorrect load on the subject. Individual calibration of the proper back posture by measuring the standing posture is also possible. Another option for posture detection could be the use of a camera to analyze the back posture of the player. Alternatively, it would also be possible to expand the tablet app to include game-like elements to increase motivation for the training sessions. Based on the posture adopted by the test subjects on the ICAROS Pro, it would be possible to display a game on the tablet in which an object controlled by the player has to fly through an obstacle course and hold a certain position for the duration of the flight. Another potential extension of the system would be the development of a parametrization system. The purpose of such a system would be to ensure that adjustments to the system, such as the addition of new training procedures, could be made without interfering with the source code. One potential approach to enhance the versatility of the system involves the implementation of a modularization design. This design could empower users to change modules, e.g., simplifying the process of recording vital parameters using alternative sensors. Another development for the system could be the use of other training equipment, such as pulleys with weights. Here, the system could help to check the correct execution of exercises using the position and orientation of the trackers.

To evaluate the training effectiveness of the system in detail, an appropriate statistical methodology needs to be utilized. A study design with a predefined outcome for testing the effectiveness needs to be defined with a corresponding sample size calculation to enable adequate effect power. To achieve generalizable results and foster comparability with other related work, it is essential to expand the recruitment of the study population to encompass a more extensive demographic range. It may further be necessary to carry out the training exercises under laboratory conditions. This study will serve as a basis for planning and conducting a more extensive follow-up study. Future evaluations of the systems may focus on potential differences in adaptation between the sexes. This could provide clarity on the observed adaptability of female subjects to the training regimen despite lower self-assigned fitness compared to the male subjects. Possible factors that could be considered are physiological factors such as height and weight, as well as BMI as a factor in the operation of the training device, or psychological backgrounds. In order to use the system in clinical rehabilitation programs, the individual target coordinates must be determined by medical professionals. This reduces incorrect loading and the potential risk of injury. When using the system with elderly people, a similar adjustment of the target coordinates is required in order to meet the specific needs of this target group and minimize risks. Furthermore, adjustments to the hardware on the ICAROS Pro are particularly useful for elderly users. The use of elastic bands can help to cushion movements and reduce the load on the body. This contributes to the safety and user-friendliness of the system and can minimize the risk of injury. In addition to these adaptations, another person should be present during training with elderly and during rehabilitation. In a further study based on the VR game, attention should be paid to the VR experience of the subjects during the recruitment process.

## 7. Conclusions

In the main study, the proposed training system was evaluated with a considerably larger and more balanced study population than in the demo initially presented in [16]. Its initial use case of rehabilitative training was adjusted with the help of a sports manager towards actual back posture training, increasing the level of exertion subjects had to handle. Yet, expressiveness of the presented statistical analyses remains limited due to a still rather small study population (n=20). One feature that emerged was the clearly visible reduction in average heartbeat rates among the female subjects as well as the reduction in average heartbeat rate of the most fit group. The now more thoroughly evaluated training system showed the potential to foster autonomous training by guiding subjects through challenging tasks and was found to be suitable for numerous further use cases, e.g., adaptation of training without use of the ICAROS Pro, or integration of different sensors to avoid the observed drift. It is also possible to extend the system for visually impaired users using audio instructions or to use the system for other training devices.

In the secondary study, the self-developed VR exergame demo received an initial evaluation based on a small study population (n=5). Feedback revealed a high level of enjoyment during training within the exergame and a generally low level of perceived exertion. The used Empatica EmbracePlus was found to be less suitable for short training sessions, as in the presented use case. This is due to a low time resolution and aggregation of heart rate and EDA measurements, only acquired every minute. Ongoing future work will thus focus on exploring alternatives for short-term vital sign monitoring. Further future work describes a possible fusion of the two systems, which then needs to be evaluated on a larger scale with a notably increased study population, based on minimum sample size calculation to achieve adequate effect power. The modularization and parametrization of the system could also offer promising possibilities to adapt the system to different training requirements.

Software components are available at: https://github.com/DaWisw/Coordination-Training-Evaluation-App-for-ICAROS-Pro (accessed on 21 February 2025). Developments of future work will be made available there as well.

## Figures and Tables

**Table 1:** Subject characteristics of the main study.

Feature	Manifestation		
Sex	M n=10 (50%)	F n=10 (50%)	All n=20 (100.00%)
	Mean ± standard deviation		
Age (years)	27.60±3.41	28.30±9.10	27.95±6.70
Height (m)	1.86±0.09	1.69±0.04	1.78±0.11
Weight (kg)	96.00±18.97	72.10±14.48	84.05±20.53
BMI	27.46±4.46	25.11±5.58	26.29±5.06
Self-assigned fitness	3.20±0.79	2.80±0.79	3.00±0.79

**Table 2:** Subject characteristics of the secondary study.

Feature	Manifestation		
Sex	M n=3 (60%)	F n=2 (40%)	All n=5 (100.00%)
	Mean ± standard deviation		
Age (years)	26.33±1.53	25.50±2.12	26.00±1.58
Height (m)	1.88±0.11	1.64±0.08	1.78±0.15
Weight (kg)	93.60±31.56	60.00±14.14	80.20±29.80
BMI	26.03±5.61	22.00±3.14	24.41±4.80
Self-assigned fitness	2.33±0.58	2.5±0.71	2.4±0.55
2-4 Prior VR experience	n=2 (66.67%)	n=2 (100.00%)	n=4 (80.00%)

**Table 3:** Survey overview with elaborations on individual questions with keys for quick identification, scopes of possible answers, and references for question origins.

Key	Question	Type	Scope	Ref.
Usability	How do you rate the VR system usability?	Likert	1–5 (Low–High)	[66]
Immersion	How do you rate the exergame immersion?	Likert	1–5 (Low–High)	[71]
Adversities	Did you experience adverse effects (e.g., dizziness, nausea)?	Binary	Yes/No (+ optional free text)	[69]
Effectiveness	How do you rate the exergame effectiveness for building muscle?	Likert	1–5 (Low–High)	–
Muscles	Which muscle groups were strained most?	Multiple	Arms, Legs, Back, Abdomen, Chest	–
Exhaustion	How do you rate the physical exhaustion during the game compared to traditional training?	Likert	1–5 (Low–High)	[70,72]
Fun	How would you rate the fun factor of the game?	Likert	1–5 (Low–High)	[73]
Motivation	How motivating do you find the game mechanics for continuous training?	Likert	1–5 (Low–High)	–
Balancing	How balanced is the game in terms of challenges and rewards?	Likert	1–5 (Poor–Good)	[74]
Graphics	How would you rate the game graphics quality?	Likert	1–5 (Poor–Good)	–
Bugs	Were there any technical problems or bugs?	Binary	Yes/No (+ optional free text)	–
Satisfaction	How satisfied are you with the exergame?	Likert	1–5 (Low–High)	–
Recommendation	Would you recommend the game to others?	Binary	Yes/No (+ optional free text)	–
Enjoyment	Did you enjoy any particular aspects?	Free text	–	–
Improvement	What could be improved for a better training experience?	Free text	–	–
Feedback	Do you have any comments or suggestions?	Free text	–	–

**Table 4:** Training pose goals and holding times.

Setting	Pose Goals with Holding Time						
Goal	1	2	3	4	5	6	7
X/Roll axis (°)	0	+10	−10	+5	−5	+15	−15
Y/Pitch axis (°)	+25	+15	+15	+20	+20	+10	+10
Time (s)	10	7	7	7	7	7	7

**Table 5:** Training session task order and pose goal alterations.

Training Session	Task	Pose Goals						
1	1	1	2	3	4	5	6	7
	2	1	5	4	7	6	3	2
	3	1	6	7	2	3	4	5
2	3	1	6	7	2	3	4	5
	2	1	5	4	7	6	3	2
	1	1	2	3	4	5	6	7
3	2	1	5	4	7	6	3	2
	1	1	2	3	4	5	6	7
	3	1	6	7	2	3	4	5

**Table 6:** Heart rate (bpm) per training session (n=20).

Training Session	Mean ± Standard Deviation	Minimum	Maximum
1	90.10±10.93	66.00	112.00
2	87.68±12.63	66.00	123.00
3	86.00±10.01	70.00	104.00

**Table 7:** Respiration rate (rpm) per training session (n=20).

Training Session	Mean ± Standard Deviation	Minimum	Maximum
1	14.78±4.75	7.06	26.92
2	13.28±3.91	6.00	21.59
3	14.05±3.33	6.72	20.48

**Table 8:** Number of incorrect back postures per training session (n=20).

Training Session	Mean ± Standard Deviation	Minimum	Maximum
1	13.87±11.57	0.00	54.00
2	15.10±11.19	0.00	39.00
3	12.95±9.51	1.00	36.00

**Table 9:** Archived game scores per training session (n=5).

Training Session	Mean ± Standard Deviation	Median	95% CI
1	26.00±3.39	25.00	[21.79,30.21]
2 ^*a*^	24.00±5.32	25.00	[17.99,31.21]
3	27.00±2.12	27.00	[24.37,29.63]

^*a*^ Caused by a single outlier in the second training. Exclusion results lead to a Mean of 26.75±2.63 [median: 27.00; 95% CI: 22.57,30.93] in the second training.

## Data Availability

The original contributions presented in this study are included in the article material. Further inquiries can be directed to the corresponding author.

## Abbreviations

The following abbreviations are used in this manuscript:

BMI	Body Mass Index
bpm	Beats per Minute
CI	Confidence Interval
CSV	Comma Separated Values
ECG	Electrocardiography
EDA	Electrodermal Activity
FDA	U.S. Food and Drug Administration
HMD	Head-Mounted Display
IR	Infrared
MR	Mixed Reality
rpm	Respirations per Minute
TCP	Transmission Control Protocol
VR	Virtual Reality

## References

[1] Weiss D., Rydland H.T., Øversveen E., Jensen M.R., Solhaug S., Krokstad S. (2018). Innovative technologies and social inequalities in health: A scoping review of the literature. PLoS ONE.

[2] Senbekov M., Saliev T., Bukeyeva Z., Almabayeva A., Zhanaliyeva M., Aitenova N., Toishibekov Y., Fakhradiyev I. (2020). The Recent Progress and Applications of Digital Technologies in Healthcare: A Review. Int. J. Telemed. Appl..

[3] Osipov V.S., Skryl T.V. (2021). Impact of Digital Technologies on the Efficiency of Healthcare Delivery. IoT in Healthcare and Ambient Assisted Living.

[4] Dal Mas F., Massaro M., Rippa P., Secundo G. (2023). The challenges of digital transformation in healthcare: An interdisciplinary literature review, framework, and future research agenda. Technovation.

[5] Frederix I., Hansen D., Coninx K., Vandervoort P., Vandijck D., Hens N., Van Craenenbroeck E., Van Driessche N., Dendale P. (2015). Effect of comprehensive cardiac telerehabilitation on one-year cardiovascular rehospitalization rate, medical costs and quality of life: A cost-effectiveness analysis. Eur. J. Prev. Cardiol..

[6] Hwang R., Bruning J., Morris N.R., Mandrusiak A., Russell T. (2017). Home-based telerehabilitation is not inferior to a centre-based program in patients with chronic heart failure: A randomised trial. J. Physiother..

[7] Pastora-Bernal J.M., Martín-Valero R., Barón-López F.J. (2017). Cost analysis of telerehabilitation after arthroscopic subacromial decompression. J. Telemed. Telecare.

[8] Fatoye F., Gebrye T., Fatoye C., Mbada C.E., Olaoye M.I., Odole A.C., Dada O. (2020). The Clinical and Cost-Effectiveness of Telerehabilitation for People With Nonspecific Chronic Low Back Pain: Randomized Controlled Trial. JMIR mHealth uHealth.

[9] Kouijzer M.M.T.E., Kip H., Bouman Y.H.A., Kelders S.M. (2023). Implementation of virtual reality in healthcare: A scoping review on the implementation process of virtual reality in various healthcare settings. Implement. Sci. Commun..

[10] Worlikar H., Coleman S., Kelly J., O’Connor S., Murray A., McVeigh T., Doran J., McCabe I., O’Keeffe D. (2023). Mixed Reality Platforms in Telehealth Delivery: Scoping Review. JMIR Biomed. Eng..

[11] Su Z., Zhang L., Lian X., Guan M. (2024). Virtual Reality–Based Exercise Rehabilitation in Cancer-Related Dysfunctions: Scoping Review. J. Med. Internet Res..

[12] Mubin O., Alnajjar F., Al Mahmud A., Jishtu N., Alsinglawi B. (2020). Exploring serious games for stroke rehabilitation: A scoping review. Disabil. Rehabil. Assist. Technol..

[13] Doumas I., Everard G., Dehem S., Lejeune T. (2021). Serious games for upper limb rehabilitation after stroke: A meta-analysis. J. Neuroeng. Rehabil..

[14] Vieira C., Ferreira da Silva Pais-Vieira C., Novais J., Perrotta A. (2021). Serious Game Design and Clinical Improvement in Physical Rehabilitation: Systematic Review. JMIR Serious Games.

[15] Damaševičius R., Maskeliūnas R., Blažauskas T. (2023). Serious Games and Gamification in Healthcare: A Meta-Review. Information.

[16] Meiszl K., Potthast T., Schulten T., Silberbach M., Wiswede D., Abbassi P., Hake L., Hussein V., de Canaviri L.K., Mirraziroudsari S.D. App-Guided ICAROS Pro Training via SteamVR Tracking 2.0 and Zephyr BioHarness 3.0. Proceedings of the 2023 IEEE 12th International Conference on Intelligent Data Acquisition and Advanced Computing Systems: Technology and Applications (IDAACS).

[17] Kuhlmann de Canaviri L., Meiszl K., Hussein V., Abbassi P., Mirraziroudsari S.D., Hake L., Potthast T., Ratert F., Schulten T., Silberbach M. (2023). Static and Dynamic Accuracy and Occlusion Robustness of SteamVR Tracking 2.0 in Multi-Base Station Setups. Sensors.

[18] Hao J., Xie H., Harp K., Chen Z., Siu K.C. (2022). Effects of Virtual Reality Intervention on Neural Plasticity in Stroke Rehabilitation: A Systematic Review. Arch. Phys. Med. Rehabil..

[19] Chen J., Or C.K., Chen T. (2022). Effectiveness of Using Virtual Reality–Supported Exercise Therapy for Upper Extremity Motor Rehabilitation in Patients With Stroke: Systematic Review and Meta-analysis of Randomized Controlled Trials. J. Med. Internet Res..

[20] Vassantachart A., Yeo E., Chau B. (2022). Virtual and Augmented Reality-based Treatments for Phantom Limb Pain: A Systematic Review. Innov. Clin. Neurosci..

[21] Ali S.G., Wang X., Li P., Jung Y., Bi L., Kim J., Chen Y., Feng D.D., Magnenat Thalmann N., Wang J. (2023). A systematic review: Virtual-reality-based techniques for human exercises and health improvement. Front. Public Health.

[22] Tortora C., Di Crosta A., La Malva P., Prete G., Ceccato I., Mammarella N., Di Domenico A., Palumbo R. (2024). Virtual reality and cognitive rehabilitation for older adults with mild cognitive impairment: A systematic review. Ageing Res. Rev..

[23] Dębska M., Polechoński J., Mynarski A., Polechoński P. (2019). Enjoyment and Intensity of Physical Activity in Immersive Virtual Reality Performed on Innovative Training Devices in Compliance with Recommendations for Health. Int. J. Environ. Res. Public Health.

[24] Feodoroff B., Konstantinidis I., Froböse I. (2019). Effects of Full Body Exergaming in Virtual Reality on Cardiovascular and Muscular Parameters: Cross-Sectional Experiment. JMIR Serious Games.

[25] Treskunov A., Gerhardt E., Nowottnik D., Fischer B., Gerhardt L., Säger M., Geiger C. (2019). ICAROSmuIti—A VR Test Environment for the Development of Multimodal and Multi-User Interaction Concepts. Proceedings of the Mensch und Computer 2019, MuC’19.

[26] Strassmann C., Arntz A., Eimler S.C. Under The (Plastic) Sea—Sensitizing People Toward Ecological Behavior Using Virtual Reality Controlled by Users’ Physical Activity. Proceedings of the 2020 IEEE International Conference on Artificial Intelligence and Virtual Reality (AIVR).

[27] Howard M.C. (2017). A meta-analysis and systematic literature review of virtual reality rehabilitation programs. Comput. Hum. Behav..

[28] Tieri G., Morone G., Paolucci S., Iosa M. (2018). Virtual reality in cognitive and motor rehabilitation: Facts, fiction and fallacies. Expert Rev. Med. Devices.

[29] Villada Castillo J.F., Montoya Vega M.F., Muñoz Cardona J.E., Lopez D., Quiñones L., Henao Gallo O.A., Lopez J.F. (2024). Design of Virtual Reality Exergames for Upper Limb Stroke Rehabilitation Following Iterative Design Methods: Usability Study. JMIR Serious Games.

[30] Davis J.C., Killen L.G., Green J.M., Waldman H.S., Renfroe L.G. (2024). Exergaming for physical activity: A systematic review. J. Am. Coll. Health J. ACH.

[31] Niehorster D.C., Li L., Lappe M. (2017). The Accuracy and Precision of Position and Orientation Tracking in the HTC Vive Virtual Reality System for Scientific Research. i-Perception.

[32] Luckett E., Key T., Newsome N., Jones J.A. (2019). Metrics for the Evaluation of Tracking Systems for Virtual Environments. Proceedings of the 26th IEEE Conference on Virtual Reality and 3D User Interfaces.

[33] Bauer P., Lienhart W., Jost S. (2021). Accuracy Investigation of the Pose Determination of a VR System. Sensors.

[34] Holzwarth V., Gisler J., Hirt C., Kunz A. (2021). Comparing the Accuracy and Precision of SteamVR Tracking 2.0 and Oculus Quest 2 in a Room Scale Setup. Proceedings of the 2021 the 5th International Conference on Virtual and Augmented Reality Simulations, ICVARS 2021.

[35] Wang Y.W., Chen C.H., Lin Y.C. Balance Rehabilitation System for Parkinson’s Disease Patients based on Augmented Reality. Proceedings of the 2020 IEEE Eurasia Conference on IOT, Communication and Engineering (ECICE).

[36] Chen L.Y., Chang L.Y., Deng Y.C., Hsieh B.C. The Rehabilitation and Assessment in Virtual Reality Game for the patient with Cognitive impairment. Proceedings of the 2020 International Symposium on Computer, Consumer and Control (IS3C).

[37] Wen H., Zhang Z., Lv C., Wang Y., Hu Y. Construction of Psychological Training System for College Students Based on Virtual Reality Technology. Proceedings of the 2022 8th International Conference on Virtual Reality (ICVR).

[38] Lima G.A.C.d., Lamounier Junior E.A., Melo B.G.R.V.d., Soares A.B., Silva A.C., Cardoso A. A Markerless Augmented Reality Application for Training Upper Limb Amputees. Proceedings of the Anais Estendidos do XXIV Simpósio de Realidade Virtual e Aumentada (SVR Estendido 2022). Sociedade Brasileira de Computação, 2022, SVR Estendido 2022.

[39] Gouveia É.R., Campos P., França C.S., Rodrigues L.M., Martins F., França C., Gonçalves F., Teixeira F., Ihle A., Gouveia B.R. (2023). Virtual Reality Gaming in Rehabilitation after Musculoskeletal Injury—User Experience Pilot Study. Appl. Sci..

[40] Jamieson A.R., Singh I., Nguyen D.V., Waghmare K.C., Singh B.G.C., Gu Y., Wijesundara M.B.J. Integrating an Assistive Soft Robotic Glove with an Immersive Virtual Reality Hand Rehabilitation Game. Proceedings of the 2023 IEEE 11th International Conference on Serious Games and Applications for Health (SeGAH).

[41] Bosch-Barceló P., Climent-Sanz C., Martínez-Navarro O., Masbernat-Almenara M., Pakarinen A., Ghosh P.K., Fernández-Lago H. (2024). A treadmill training program in a gamified virtual reality environment combined with transcranial direct current stimulation in Parkinson’s Disease: Study protocol for a randomized controlled trial. PLoS ONE.

[42] De Fazio R., Mastronardi V.M., De Vittorio M., Visconti P. (2023). Wearable Sensors and Smart Devices to Monitor Rehabilitation Parameters and Sports Performance: An Overview. Sensors.

[43] Al-Ayyad M., Owida H.A., De Fazio R., Al-Naami B., Visconti P. (2023). Electromyography Monitoring Systems in Rehabilitation: A Review of Clinical Applications, Wearable Devices and Signal Acquisition Methodologies. Electronics.

[44] Hintz C., Presley D.M., Butler C.R. (2023). Heat stroke burden and validity of wearable-derived core temperature estimation during elite military training. Physician Sportsmed..

[45] Romagnoli S., Sbrollini A., Nocera A., Morettini M., Gambi E., Bondi D., Pietrangelo T., Verratti V., Burattini L. Sport DB 2.0: A New Database of Data Acquired by Wearable and Portable Devices While Practicing Sport. Proceedings of the 2023 Computing in Cardiology Conference (CinC), Computing in Cardiology, CinC2023.

[46] Sbrollini A., Marcantoni I., Lunghi T., Morettini M., Burattini L. (2024). Cardiorespiratory DB: Collection of cardiorespiratory data acquired during normal breathing, deep breathing and breath holding. Data Brief.

[47] Citarelli F. (2022). Metrological Characterization of a Wearable Device and a Machine Learning Algorithm for Assessment Of Sport Activities Performances. Master’s Thesis.

[48] Cosoli G., Antognoli L., Panni L., Scalise L. The Indirect Estimation of Breathing Rate through Wearables: Experimental Study and Uncertainty Analysis through Monte Carlo Simulation. Proceedings of the 2023 IEEE International Symposium on Medical Measurements and Applications (MeMeA).

[49] Panni L., Cosoli G., Antognoli L., Scalise L. (2024). Measurement of respiratory rate with cardiac belt: Metrological characterization. Meas. Sens..

[50] Ray I., Liaqat D., Gabel M., de Lara E. Skin tone, Confidence, and Data Quality of Heart Rate Sensing in WearOS Smartwatches. Proceedings of the 2021 IEEE International Conference on Pervasive Computing and Communications Workshops and other Affiliated Events (PerCom Workshops).

[51] Gashi S., Min C., Montanari A., Santini S., Kawsar F. (2022). A multidevice and multimodal dataset for human energy expenditure estimation using wearable devices. Sci. Data.

[52] Cosoli G., Antognoli L., Scalise L. (2023). Wearable Electrocardiography for Physical Activity Monitoring: Definition of Validation Protocol and Automatic Classification. Biosensors.

[53] Cosoli G., Antognoli L., Panni L., Scalise L. (2024). Indirect Estimation of Breathing Rate Using Wearable Devices. IEEE Trans. Instrum. Meas..

[54] Denyer H., Ramos-Quiroga J.A., Folarin A., Ramos C., Nemeth P., Bilbow A., Woodward E., Whitwell S., Müller-Sedgwick U., Larsson H. (2022). ADHD Remote Technology study of cardiometabolic risk factors and medication adherence (ART-CARMA): A multi-centre prospective cohort study protocol. BMC Psychiatry.

[55] Stojchevska M., de Brouwer M., Courteaux M., Steenwinckel B., van Hoecke S., Ongenae F. (2024). Unlocking the potential of smartphone and ambient sensors for ADL detection. Sci. Rep..

[56] Badawi A., Elmoghazy S., Choudhury S., Elgazzar S., Elgazzar K., Burhan A. (2024). A Novel Multimodal System to Predict Agitation in People with Dementia Within Clinical Settings: A Proof of Concept. arXiv.

[57] Empatica EmbracePlus|The World’s Most Advanced Smartwatch for Continuous Health Monitoring. 25 November 2024. https://www.empatica.com/embraceplus/.

[58] Gerboni G., Comunale G., Chen W., Lever Taylor J., Migliorini M., Picard R., Cruz M., Regalia G. (2023). Prospective clinical validation of the Empatica EmbracePlus wristband as a reflective pulse oximeter. Front. Digit. Health.

[59] Mologne M.S., Yamamoto T., Viggiano M., Blatney A.E., Lechner R.J., Nguyen T.H., Doyle A., Farrales J.P., Neufeld E.V., Dolezal B.A. (2024). Field-based fitness measures improve via an immersive virtual reality exergaming platform: A randomized controlled trial. Front. Virtual Real..

[60] Lu Y., Ota K., Dong M. (2024). An Empirical Study of VR Head-Mounted Displays Based on VR Games Reviews. Games Res. Pract..

[61] Du Q., Song Z., Jiang H., Wei X., Weng D., Fan M. (2024). LightSword: A Customized Virtual Reality Exergame for Long-Term Cognitive Inhibition Training in Older Adults. Proceedings of the CHI Conference on Human Factors in Computing Systems.

[62] Olberding H., Vetter M. (2023). Analysis of Cartographic Symbols as Visual Support in Interactive VR Geovisualizations. Proc. ICA.

[63] Polat M.D., Izzetoglu K., Aksoy M.E., Kitapcioglu D., Usseli T., Yoner S.I., Schmorrow D.D., Fidopiastis C.M. (2023). Cognitive Load Quantified via Functional Near Infrared Spectroscopy During Immersive Training with VR Based Basic Life Support Learning Modules in Hostile Environment. Proceedings of the Augmented Cognition.

[64] ICAROS GmbH (2022). ICAROS Tablet Holder Universal. Technical Report, ICAROS GmbH, Martinsried, Germany. https://www.icaros.com/fileadmin/user_upload/ICAROS_Tablet_Holder_Universal_-_Installation__Home__Pro__Health_.pdf.

[65] Sbrollini A., Bondi D., Romagnoli S., Morettini M., Marcantoni I., Pietrangelo T., Verratti V., Burattini L. Segmented-Beat Modulation Method-Based Procedure for Extraction of Electrocardiogram-Derived Respiration from Data Acquired by Wearable Sensors During High-Altitude Activity. Proceedings of the 2022 Computing in Cardiology Conference (CinC). Computing in Cardiology, CinC2022.

[66] Brooke J. (1996). SUS: A ’Quick and Dirty’ Usability Scale. Usability Evaluation In Industry.

[67] Laugwitz B., Held T., Schrepp M. (2008). Construction and Evaluation of a User Experience Questionnaire. HCI and Usability for Education and Work.

[68] Schubert T., Friedmann F., Regenbrecht H. (2001). The Experience of Presence: Factor Analytic Insights. Presence Teleoperators Virtual Environ..

[69] Kennedy R.S., Lane N.E., Berbaum K.S., Lilienthal M.G. (1993). Simulator Sickness Questionnaire: An Enhanced Method for Quantifying Simulator Sickness. Int. J. Aviat. Psychol..

[70] Borg G.A. (1982). Psychophysical bases of perceived exertion. Med. Sci. Sport. Exerc..

[71] Berkman M.İ., ÇATAK G. (2021). I-group presence questionnaire: Psychometrically revised English version. Mugla J. Sci. Technol..

[72] Williams N. (2017). The Borg Rating of Perceived Exertion (RPE) scale. Occup. Med..

[73] Phan M.H., Keebler J.R., Chaparro B.S. (2016). The Development and Validation of the Game User Experience Satisfaction Scale (GUESS). Hum. Factors.

[74] Sweetser P., Wyeth P. (2005). GameFlow. Comput. Entertain..

